# Influenza vaccine uptake among community-dwelling Italian elderly: results from a large cross-sectional study

**DOI:** 10.1186/1471-2458-11-207

**Published:** 2011-04-01

**Authors:** Carlos Chiatti, Pamela Barbadoro, Giovanni Lamura, Lucia Pennacchietti, Francesco Di Stanislao, Marcello M D'Errico, Emilia Prospero

**Affiliations:** 1 Department of Biomedical Sciences, Section of Hygiene and Public Health, Polytechnic University of the Marche Region, via Tronto, 10/a, Torrette di Ancona, 60020, Italy; 2 Department of Gerontological Research, Italian National Research Centre on Ageing - I.N.R.C.A., Via Santa Margherita 5, Ancona, 60100, Italy

**Keywords:** influenza vaccine, older people, Italy

## Abstract

**Background:**

Flu vaccination significantly reduces the risk of serious complications like hospitalization and death among community-dwelling older people, therefore vaccination programmes targeting this population group represent a common policy in developed Countries. Among the determinants of vaccine uptake in older age, a growing literature suggests that social relations can play a major role.

**Methods:**

Drawing on the socio-behavioral model of Andersen-Newman - which distinguishes predictors of health care use in predisposing characteristics, enabling resources and need factors - we analyzed through multilevel regressions the determinants of influenza immunization in a sample of 25,183 elderly reached by a nationally representative Italian survey.

**Results:**

Being over 85-year old (OR = 1.99; 95% CI 1.77 - 2.21) and suffering from a severe chronic disease (OR = 2.06; 95% CI 1.90 - 2.24) are the strongest determinants of vaccine uptake. Being unmarried (OR = 0.81; 95% CI 0.74 - 0.87) and living in larger households (OR = 0.83; 95% CI 0.74 - 0.87) are risk factors for lower immunization rates. Conversely, relying on neighbors' support (OR = 1.09; 95% CI 1.02 - 1.16) or on privately paid home help (OR = 1.19; 95% CI 1.08 - 1.30) is associated with a higher likelihood of vaccine uptake.

**Conclusions:**

Even after adjusting for socio-demographic characteristics and need factors, social support, measured as the availability of assistance from partners, neighbors and home helpers, significantly increases the odds of influenza vaccine use among older Italians.

## Background

Many studies support the hypothesis that flu vaccination significantly reduces the risk of serious complications, e.g. hospitalization for pneumonia or influenza, and of death among community dwelling elderly persons [1], and suggest that annually repeated vaccine is essential for maintaining vaccine efficacy over time [2]. Despite the methodological quality of studies assessing vaccine efficacy is controversial [3,4], vaccination programmes targeted at people aged 65 and over represent a common policy in industrialized Countries, and their importance has been recently highlighted [5].

Recent studies suggested that, among the elderly, an important role in predicting immunization is represented also by the availability [6,7] and quality of social support [8] on which the older person can count. The primary aim of this study is to examine whether the likelihood of vaccine uptake among older Italians is influenced by the level of social support they receive. The secondary aim is to elucidate other factors that may constitute potential predictors of elderly influenza vaccination in the Country.

## Methods

The Multi-Purpose survey "Health status of the population and use of health services in Italy" [9] carried out by the Italian National Institute of Statistics (ISTAT), aimed at investigating a variety of aspects concerning the health of the population and the patterns of health care use. The last edition of this survey, carried out between December 2004 and September 2005, gathered data on 25,183 persons aged 65 and older, representative in terms of age and gender of the Italian elderly population [10]. Each person participating to the survey completed first a self-administered questionnaire, and then had a face-to-face interview with ISTAT data collectors. In case the index person was cognitively impaired or not available, the interview was administered to another family member who responded in his/her place. The final dataset was openly available by means of a formal request to ISTAT.

### Dependent and independent variables

As a dependent variable, we used the reply (yes/no) to the question: "Did you get vaccinated against influenza in the last 12 months?" Self-report has been shown to be a highly accurate way of assessing past vaccination behavior in elderly populations [11].

We selected independent variables drawing on the socio-behavioral model of Andersen-Newman, which distinguishes the explanatory factors of health care use in predisposing characteristics, enabling resources, and need factors [12,13]. In this study, we focused primarily on the role of enabling resources, i.e. those variables related to the social supports available to the older person. The ISTAT questionnaire included several items to measure the level of social support benefited by the older person: we considered marital status (married or cohabiting vs. single, widowed, divorced or separated), the availability of informal help from family, friends or neighbors, and the support received from privately paid domestic workers (distinguishing between home helper, i.e. those workers providing support with the housework, and carers, i.e. those providing assistance to the older person with ADL and IADL limitations [14]). Predisposing characteristics were retrieved for each subject: gender, age, education, self-report household wealth and smoking status. Need factors were taken into account by using self-rated health status and the presence of chronic illnesses.

### Statistical analysis

Bivariate analysis was performed to analyze the distribution of the outcome and the exposure variables in the sample. Since the Italian NHS has undergone a federalist reform, and regions have some degree of independence in organizing local health services, persons living in same areas tend to experience similar access to health care. Therefore, we used multilevel regression models that consider the connection of the outcome variable among people from the same area, to obtain accurate statistical estimates of vaccine predictors [15]. In the models, the geographical area of residence (categorized as North-East, North-West, Centre, South, and Islands) was included as random-effect parameter. Covariates were included into the models with a forward stepwise procedure. An indicator variable for respondent type was included in the regressions, as index and proxy respondent data were pooled together. Model 1 includes only variables related to the enabling resources, while other two models additionally control for predisposing characteristics (Model 2) and need factors (Model 3). Analyses were performed using STATA, version 10.1 (Stata Corp., College Station, TX, 2007) [16].

## Results

Mean age in the sample is 74.7 years (SD ± 7.1). 57.6% (n = 14,515) of the subjects are women and 56.9% (n = 14,318) are married or cohabiting (see Table 1 for further socio-demographic characteristics of the sample). Index subjects completed the interview in 86.6% (n = 21,813) and proxy respondents in 6.8% of cases (n = 1,708), while the remaining 4.9% (n = 1,239) required assistance from other family members to provide information on exposures. Information on respondent type was missing in 1.7% of the sample (n = 482).

**Table 1:** Immunization rates according to main sociodemographic characteristics among a representative sample of older Italians (n = 25,183)

			Vaccination rate	
			(%)	**p**^**C**^
**Enabling resources**	*Marital status*	Married/Cohabiting (n = 14,318)	62.3	0.286
		Single/Widowed (n = 10,865)	63.0	
	*Help from family*	No (n = 3,773)	60.5	0.003
		Yes (n = 21,410)	63.0	
	*Help from friends*	No (n = 12,731)	63.3	0.022
		Yes (n = 12,452)	61.9	
	*Help from neighbours*	No (n = 12,754)	61.7	0.004
		Yes (n = 12,429)	63.5	
	*Household size*	- Single person (n = 16,977)	63.8	<0.001
		- Couple (n = 11,953)	64.6	
		- 3-5 persons (n = 5,865)	57.3	
		- More than 6 persons (n = 388)	59.5	
	*Private domestic worker: home helper*	No (n = 22,335)	61.9	<0.001
		Yes (n = 2,848)	67.9	
	*Private domestic worker: home carer*	No (n = 24,169)	62.1	<0.001
		Yes (n = 1,014)	73.6	

**Predisposing characteristics**	*Gender*	Male (n = 10,668)	61.6	0.008
		Female (n = 14,515)	63.3	
	*Age (in years)*	- 65 - 74 (n = 13,667)	55.4	<0.001
		- 75 - 84 (n = 9,098)	70.5	
		- 85 + (n = 2,418)	73.5	
	*Education*^A^	- Low (n = 3,818)	66.5	<0.001
		- Medium-low (n = 13,828)	63.3	
		- Medium-high (n = 4,674)	59.4	
		- High (n = 2,863)	59.3	
	*Self-reported household wealth*	- Low (n = 923)	65.2	0.002
		- Medium-low (n = 8,077)	64.0	
		- Medium-high (n = 15,378)	61.7	
		- High (n = 805)	63.7	
	*Smoking status*	Smoker (n = 22,996)	52.0	<0.001
		Non smoker (n = 2,187)	63.6	

**Need factors**	*Chronic diseases*^B^	- No (n = 4,191)	47.0	<0.001
		- Yes, mild (n = 9,891)	60.1	
		- Yes, severe (n = 11,101)	70.7	
	*Self-reported health status*	- Good (n = 5,768)	50.3	<0.001
		- Fair (n = 14,292)	64.5	
		- Bad (n = 5,123)	71.1	
**Survey Respondent**	*Respondent type*	- Index subject (n = 21,813)	62.8	<0.001
		- Index subject with help (n = 1,239)	68.0	
		- Proxy respondent (n = 1,708)	55.7	
		*Total sample *(n = 25,183)	62.6	

^A ^Educational level was categorized as follows: Low (no title), Medium-Low (primary school degree), Medium-Upper (intermediate degree), Upper (high school, bachelor or higher); ^B ^We categorized as severe the following chronic diseases: diabetes, cardiac disease, stroke, neoplasm, chronic pulmonary obstructive disease, Alzheimer and other forms of dementia and cirrhosis. Other chronic conditions have been categorized as mild. ^C ^Chi square test result for the association between vaccine uptake and individuals' characteristics.

Overall, 62.6% (n = 25,183) of the sample reported to be vaccinated against seasonal flu. The analysis of vaccine uptake rates stratified according to study variables reveals significant differences between the various subgroups of the sample (Table 1). A higher immunization rate was observed among those older persons relying on the support provided by a private home helper (67.9 vs. 61.9; p < 0.001) or carer (73.6 vs. 62.1; p < 0.001). Among the enabling resources, no association at bivariate level could be found only for marital status (married vs. single, widowed and separated/divorced persons). Considering predisposing variables, immunization rate is higher among very old (85+ years) (73.5%), less educated (66.5%) and less wealthy subjects (65.5%). With regard to need factors, crude rates suggest that those suffering from a severe chronic disease (70.7%) and reporting being in bad health conditions (71.1%) are more frequently immunized against seasonal influenza.

As what concern the fixed effects investigated in the multilevel regression, all enabling resources (model 1) are significantly associated with vaccine uptake, with the exception of marital status (Table 2). In particular, employing a private home carer (OR = 1.52; 95% CI 1.31 - 1.76) or a home helper (OR = 1.26; 95% CI 1.16 - 1.38) is associated to higher vaccination rates. Conversely those living in household with 3 up to 5 persons have a lower likelihood of being immunized (OR = 0.79; 95% CI 0.72- 0.86).

**Table 2:** Results of multilevel regression models for estimated of factors associated with influenza vaccine uptake in the previous 12 months among a representative sample of older Italians (n = 24,760)

	Model 1 *(Enabling resources)*	Model 2 (*Model 1 +* *Predisposing characteristics*)	Model 3 (*Model 2 +* *Need factors*)
	OR (95% CI)	OR (95% CI)	OR (95% CI)
**Fixed effects**			
*Marital status*, *not married *(n = 10,670)	0.97 (0.90 - 1.05)	0.82 (0.76 - 0.89)*	0.81 (0.74 - 0.87)*
*Help availability from:*			
- Family (n = 21,040)	1.09 (1.01 - 1.17)*	1.02 (0.94 - 1.10)	1.02 (0.95 - 1.10)
- Friends (n = 12,221)	0.90 (0.85 - 0.96)*	0.96 (0.90 - 1.02)	1.00 (0.94 - 1.06)
- Neighbors (n = 12,212)	1.12 (1.05 - 1.19)*	1.08 (1.02 - 1.15)*	1.08 (1.02 - 1.15)*
*Household size*			
- Single person (n = 6,844)	1	1	1
- Couple (n = 11,774)	1.05 (0.96 - 1.15)	1.07 (0.98 - 1.17)	1.06 (0.97 - 1.16)
- 3-5 persons (n = 5,760)	0.79 (0.72 - 0.86)*	0.84 (0.76 - 0.92)*	0.83 (0.76 - 0.92)*
- More than 6 persons (n = 382)	0.85 (0.69 - 1.07)	0.84 (0.67 - 1.05)	0.83 (0.66 - 1.04)
*Privately paid help *(n = 2,804)	1.26 (1.16 - 1.38)*	1.24 (1.13 - 1.35)*	1.19 (1.08 - 1.30)*
*Privately paid carer *(n = 991)	1.52 (1.31 - 1.76)*	1.22 (1.05 - 1.42)*	1.08 (0.93 - 1.26)

*Gender, female *(n = 14,285)		1.01 (0.95 - 1.07)	0.99 (0.93 - 1.05)
*Age*			
- 65-74 (n = 13,434)		1	1
- 75-84 (n = 8,960)		1.88 (1.77 - 1.99)*	1.72 (1.62 - 1.83)*
- 85+ (n = 2,366)		2.23 (2.01 - 2.48)*	1.99 (1.77 - 2.21)*
*Educational level*^A^		0.94 (0.90 - 0.97)*	0.96 (0.93 - 0.99)*
*Self-reported household wealth*^B^		0.95 (0.91 - 0.99)*	1.03 (0.98 - 1.08)
*Smoking status, smoker *(n = 2,149)		0.71 (0.65 - 0.78)*	0.73 (0.66 - 0.80)*

*Chronic diseases*^C^			
- No (n = 4,105)			1
- Mild (n = 9, 768)			1.46 (1.35 - 1.58)*
- Severe (n = 10,887)			2.06 (1.90 - 2.24)*
*Self-reported health status*			
- Good (n = 5,679)			0.70 (0.65 - 0.75)*
- Fair (n = 14,054)			1
- Bad (n = 5,027)			1.08 (1.00 - 1.17)*

*Respondent type*			
- Index subject (n = 21,813)	1	1	1
- Index subject with help (n = 1,239)	1.24 (1.09 - 1.40)*	0.99 (0.87 - 1.13)	0.87 (0.76 - 0.99)*
- Proxy respondent (n = 1,708)	0.78 (0.70 - 0.86)*	0.75 (0.67 - 0.83)*	0.73 (0.65 - 0.81)*

**Random effects parameter**			
Area of residence, Variance estimated	0.13 ± 0.4*	0.13 ± 0.4*	0.13 ± 0.4*
Intraclass correlation (ρ)	0.00502	0.00498	0.00530

OR = Odds Ratio; CI = Confidence Interval. ^A ^Educational level was treated as an ordinal variable; ^B ^Self-reported household wealth was treated as an ordinal variable ^C ^We categorized as severe the following chronic diseases: diabetes, cardiac disease, stroke, neoplasm, chronic pulmonary obstructive disease, Alzheimer and other forms of dementia and cirrhosis. Other chronic conditions have been categorized as mild. * Statistical significance below 0.05 level.

When considering also predisposing characteristics (model 2), older age is the strongest predictor of vaccine uptake. Both a higher education (OR = 0.94; 95% CI 0.90 - 0.97), higher household wealth (OR = 0.95; 95% CI 0.91 - 0.99) and being a smoker (OR = 0.71; 95% CI 0.65 - 0.78) reduce the likelihood of vaccination. In model 2, as a consequence of the adjustment made by predisposing characteristics, help availability from family and friends have no longer influence on influenza immunization while, conversely, not being married becomes a risk factor for undervaccination (OR = 0.82; 95% CI 0.76 - 0.89).

When inserting variables related to need factors (model 3), having a mild (OR = 1.46; 95% CI 1.35 - 1.58) or a severe chronic diseases (OR = 2.06; 95% CI 1.90 - 2.24) and bad self-rated health (OR = 1.08; 95% CI 1.00 - 1.17) are predictors of vaccine uptake, while reporting good health (OR = 0.70; 95% CI 0.66 - 0.75) decreases the odds of influenza immunization. In this last model, employing a private carer no longer determines a higher vaccine uptake.

In model 3, the likelihood of vaccination was lower among both older people who replied through a proxy respondent (OR = 0.73 95% CI 0.65 - 0.81) or thanks to the help from another family member (OR = 0.87 95% CI 0.76 - 0.99).

## Discussion

Results concerning vaccine uptake rates are consistent with the Italian Ministry of Health's estimates, according to which vaccine coverage among the older population was about 63.4% in 2003/2004 and 66.6% in 2004/2005, thus confirming the representativeness of the sample [17].

Our results suggest that social support contributes to explain influenza vaccine use among the older population, although also other factors have an important role in predicting influenza immunization. The multivariate analysis that we performed in three different steps (i.e. 1. considering enabling resources *per se*; 2. inserting predisposing characteristics; and 3. considering need factors) allowed us to assess the influence of social support, controlling for possible confounding associations on vaccine uptake.

The geographical area of residence, treated as a random effect parameter, had a significant effect in all three models, this justifying the choice of using multilevel rather than traditional logistic regressions.

Interestingly, even if marital status and vaccine are not associated at a bivariate level, after adjusting for age, we found that married elderly have a significant immunization edge compared to their non-married counterparts. These findings are coherent with other studies asserting that married older people are more frequently using preventive services [18]. In addition, the lower likelihood of older people living in larger household (from 3 to 5 persons) to be immunized confirms that older people living in couples are those more frequently vaccinated.

Good relations with friends no longer predict a lower vaccination uptake in model 2, hinting that a confounding caused by age takes place at bivariate analysis, since younger old people tend at the same time to be less vaccinated and to be in stronger friendship networks.

We noticed also that people with good relations with their neighbors (i.e. who can count on their help in case of need) have higher odds of vaccination. A similar effect was acknowledged in the US and UK using area-based indexes of deprivation, where lower uptake has been recorded in socially deprived areas [19-21].

As what concerns the role of the formal support received by the older persons in their households, we acknowledged that the association between immunization and private care work is only the result of a confounding caused by age (in model 2, the OR of vaccination for those privately hiring a care worker decreases from 1.57 to 1.22) and health conditions (in model 3, this OR is no longer significant). This occurs because in Italy those who rely more frequently on private care work are the older people with severe health problems, which, in turn, are already those more frequently immunized.

Conversely, in all the models an independent association between influenza vaccine uptake and the employment of a private home helper was observed. The process of "bridging" has been proposed to explain the higher odds of using health care services among older people relying on family caregivers; in this model, it is hypothesized that informal carers may act as advocate in acquiring formal help services for their older relatives [22]. Since family support in Italy is declining and older people increasingly rely on the care provided in their household by private workers [23], our findings suggest that a similar process of bridging may occur also in the case of privately-purchased support. However, further specific analyses on this issue are required.

The effects of predisposing characteristics and need factors are consistent with previous studies. Older age is a strong predictor of being vaccinated in different national contexts [7,24,25], as this is clearly shown also by the ISTAT data. In line with existing evidence for the Italian context [24,26], neither a positive nor a negative effect for gender was found in our study.

The role of socioeconomic factors is discussed controversially in the literature. In the US and UK, higher occupational status and education [20,25] and good housing conditions [6] are predictors of vaccination uptake. Our study, using two traditional indicators of socioeconomic positions like educational level and self-reported household wealth, shows that people in higher social position have less chances of being immunized. These results seem to confirm that in Italy, differently from what occurs in other Countries, seasonal influenza vaccination programmes are equity-oriented [26,27]. Finally, the role of need variables in predicting vaccine uptake is confirmed by our findings, which are consistent with studies establishing that worse physical conditions (as for instance suffering from a chronic disease) result strongly associated with immunization [28,29]. Reporting being in good health in our study is a risk factor for a lower vaccine uptake, suggesting that in Italy like in other Countries [30], this constitutes one of the most common reasons for non uptake.

The likelihood of flu vaccination is lower when the interview is carried out through a proxy respondent or thanks to the help from another family member (although this occurs only in the final model, when adjusting also for health-related variables). In order to understand these predictors, we stratified our sample according to respondent type, finding that subjects requiring assistance during the interview are mostly very old (mean age 80.7 years), women (62.6%) and in bad health status (67.2% reported severe chronic conditions and 50.1% bad self-rated health). This socio-demographic profile can explain the progressive changes in this exposure variable effects observed in the three regression models, but not why the inability to respond autonomously represents a risk factor for non vaccination. Plausibly, this could relate to an underreporting bias, as suggested by Nelson et al. [31], occurring when health care use is reported by a proxy respondent. Although more focused studies are required in this respect, our results strongly suggest that it is necessary to take into account the respondent type variable when analyzing health care use patterns in older populations.

### Limits of the study and policy implications

A major strength of the study is the size of the sample and its representativeness of the Italian older population; furthermore, the questionnaire used by the ISTAT is based on well-validated instruments and received several improvements since the first edition in 1980.

Nevertheless, this study has some limitations that must be considered. First, since ISTAT survey is a cross-sectional one, we were able to assess only epidemiological associations between variables, rather than causal pathways. Secondly, the study is an observational one, and even if we included in the models important covariates, residual confounding may have still influenced results. During statistical analysis we took into consideration several variables not presented in the final models, such as specific chronic diseases (e.g. diabetes, COPD), hospital and GP use, and other health related habits (e.g. physical exercises, diets). However, no meaningful changes occurred in our results. A third limitation relates to the lack of stronger standardized measure of social support, as for instance the Multidimensional Scale of Perceived Social Support or other measures of caregivers' burden. Nonetheless, most of the social risk factors identified from our study are basic socio-demographic characteristics (e.g. marital status, household size, employment of private domestic workers) that can be easily assessed by health professionals responsible for vaccine administration.

## Conclusions

In our study, we found several predictors of flu vaccination related both to demographic characteristics (e.g. older age) and health status (e.g. suffering from a chronic disease). Even when controlling for several confounders, variables reflecting the strength of social support - measured in terms of assistance made available by partners, neighbors and home helpers - increase significantly the odds of influenza vaccine use among older Italians.

In Italy, the coverage level of flu vaccination remains well below that recommended by the WHO [32] (62.6% in the sample vs. 75.0%). If further increase in the coverage wants to be reached, previous studies demonstrated the effectiveness among older population of specifically targeted interventions (for instance special vaccination hours for elderly people, television advertising, and GP's reminder programmes) [33]. In this regard, the predictors identified by this study should be taken into account in order to identify the groups of population which such interventions should primarily address.

## Competing interests

The authors declare that they have no competing interests.

## Authors' contributions

CC, PB, GL and EP conceived and designed the study. CC, PB, GL and LP performed most of the analyses on collected data, suggested and elaborated most of data interpretation criteria, and drafted the manuscript. CC and PB performed most of the final statistical analyses. FDS, MD, GL and EP relevantly contributed to manuscript revision. EP, FDS and MD were involved in critical evaluation, interpretation and discussion of data, as well as in critical revision of the final manuscript and its revision. All authors read and approved the final version of the present manuscript.

## Pre-publication history

The pre-publication history for this paper can be accessed here:

http://www.biomedcentral.com/1471-2458/11/207/prepub
